# OGG1 aggravates renal ischemia–reperfusion injury by repressing PINK1‐mediated mitophagy

**DOI:** 10.1111/cpr.13418

**Published:** 2023-02-14

**Authors:** Fan Zhao, Jiefu Zhu, Mingjiao Zhang, Yanwen Luo, Yuzhen Li, Lang Shi, Jing Huang, Halinuer Shadekejiang, Shengyu Dong, Xiongfei Wu

**Affiliations:** ^1^ Department of Nephrology Renmin Hospital of Wuhan University Wuhan Hubei China; ^2^ Department of Organ Transplantation Renmin Hospital of Wuhan University Wuhan Hubei China

## Abstract

Renal ischemia–reperfusion injury (IRI) is mainly responsible for acute kidney injury for which there is no effective therapy. Accumulating evidence has indicated the important role of mitophagy in mitochondrial homeostasis under stress. OGG1 (8‐oxoguanine DNA glycosylase) is known for functions in excision repair of nuclear and mitochondrial DNA. However, the role of OGG1 in renal IRI remains unclear. Herein, we identified OGG1, induced during IRI, as a key factor mediating hypoxia‐reoxygenation‐induced apoptosis in vitro and renal tissue damage in a renal IRI model. We demonstrated that OGG1 expression during IRI negatively regulates mitophagy by suppressing the PINK1/Parkin pathway, thereby aggravating renal ischemic injury. OGG1 knockout and pharmacological inhibition attenuated renal IRI, in part by activating mitophagy. Our results elucidated the damaging role of OGG1 activation in renal IRI, which is associated with the regulatory role of the PINK1/Parkin pathway in mitophagy.

## Graphical abstract

Schematic diagram of molecular model proposed in this study. OGG1 inhibits the clearance of damaged mitochondria under IR conditions, leading to injury of renal tubular epithelial cells. OGG1 expression is upregulated in renal tubular epithelial cells under IR conditions. OGG1 inhibits PINK1 transport to mitochondria, thereby inhibiting the activation of PINK1/Parkin mitophagy pathway, reducing the clearance of damaged mitochondria, and aggravating mitochondrial functional damage, including increased mitochondrial ROS production, decreased mitochondrial membrane potential, and decreased SOD and ATP levels. Further recruitment of Bax and downregulation of Bcl2 promote apoptosis and aggravate the injury of renal tubular epithelial cells induced by IR.

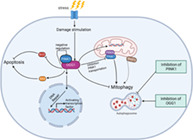

## 1 INTRODUCTION

Renal ischemia–reperfusion injury (IRI) is a secondary injury caused by the restoration of blood or oxygen supply to ischemic kidney tissue, which occurs mostly secondary to kidney transplantation, hemorrhagic shock, sepsis, and other diseases.^1^ The underlying pathophysiological mechanism of renal IRI involves mitochondrial metabolic disorders, oxidative stress, calcium overload, inflammatory reaction and apoptosis,^2^, ^3^ which is manifested by decreased mitochondrial membrane potential (MMP) and increased mitochondrial fission.^4^, ^5^, ^6^ Although the elucidation of the mechanism of renal IRI has progressed in recent years, there is currently no specific treatment for renal IRI. The oxidative stress response during ischemia–reperfusion (IR) is a key pathogenetic mechanism of renal IRI. Guanine in DNA has the lowest redox potential and is most likely to be oxidized to 8‐oxguanine (8‐oxoG), which is used as a marker of DNA oxidation. 8‐oxoG is mutagenic, and the oxidized bases are mainly removed by the base excision repair pathway.^7^ 8‐Oxoguanine DNA glycosylase (OGG1) is an important DNA repair enzyme that specifically removes 8‐oxoG from DNA duplexes during the process of base excision repair and is involved in the repair of oxidation‐induced damage of DNA.^8^

Some studies have suggested that the upregulation of OGG1 expression in brain IRI attenuates brain IRI.^9^ In renal IRI, OGG1 expression is upregulated in renal tissue at the dermomedullary junction and outer medulla.^10^ However, the role and mechanism of OGG1 in renal IRI, remain unclear.

Mitophagy is the core of mitochondrial quality control and selectively removes damaged or dysfunctional mitochondria to maintain mitochondrial homeostasis.^11^ Currently, there are two major pathways of mitophagy, which are, respectively, mediated by the PTEN‐induced kinase 1 (PINK1)‐parkin RBR E3 ubiquitin protein ligase (PARK2) pathway and mitochondria receptor‐mediated mitophagy pathways.^12^ The recent studies demonstrated that the activation of PINK1/PARK2 mitophagy pathway plays a protective role in renal IRI models.^13^ Activation of mitophagy mediated by FUN14 domain‐containing protein 1 is helpful to maintain mitochondrial homeostasis and play a role in renal protection during IRI.^14^ Some studies have confirmed that OGG1 regulates mitophagy by binding to genes or proteins in the mitophagy pathway.^15^, ^16^ During renal IRI, the expression of OGG1 is upregulated at the dermomedullary junction and outer medulla of renal tissue, and 8‐oxoG accumulates in mitochondrial DNA rather than in nuclear DNA.^10^ Therefore, we hypothesized that OGG1 is involved in the pathogenesis of renal IRI by regulating mitophagy. However, the effects of OGG1 on mitochondrial function in renal IRI remain unclear. In this study, we established OGG1 knockout (KO) mice to study the role and mechanism of action of OGG1 in renal IRI. We found that OGG1 KO or use of OGG1 inhibitors alleviated renal IRI. We propose that OGG1 may affect mitophagy and participate in renal IRI through the regulation of PINK1.

## 2 MATERIALS AND METHODS

### 2.1 Antibodies and special reagents

The antibodies used in this study were as follows: anti‐OGG1 (NB100‐106) and anti‐PINK1 (8E10.1D6) from Novus (Novus Biologicals), anti‐Parkin (sc‐32282) and anti‐TOM20 (sc‐17764) from Santa Cruz, anti‐LC3 (12741), and anti‐BCL2 (3498 S) from CST, anti‐COXIV (ab202554) from Abcam, anti‐α‐tubulin (11224‐1‐AP), anti‐BAX (60267‐1‐Ig), and anti‐cyclophilin B (11607‐1‐AP) from ProteinTech. All the secondary antibodies were purchased from Thermo Fisher Scientific. The specific inhibitor, Th5487, of OGG1 (HY‐125276) was purchased from Med Chem Express.

### 2.2 Cell culture and hypoxia‐reoxygenation treatment of BUMPTs

The BUMPT (mouse proximal tubular) cell line was originally obtained from Dr. William Lieberthal and Dr. John Shwartz at Boston University. BUMPT cells were cultured in Dulbecco's modified Eagle's medium (Thermo Fisher Scientific) with 10% fetal bovine serum (Gibco), penicillin (100 U/mL), and streptomycin (100 μg/mL) in a humidified atmosphere of 5% CO_2_ at 37°C. To construct the BUMPTs renal hypoxia‐reoxygenation (HR) model, cells were cultured in serum‐free medium and exposed to hypoxia in a tri‐gas incubator (94% N_2_, 5% CO_2_, and 1% O_2_) for 24 h followed by 4 h of reoxygenation (95% air and 5% CO2). To inhibit OGG1, 10 μM Th5487 or 0.1% dimethyl sulfoxide was used according to the indicated experimental scheme for the appropriate periods.

### 2.3 Short hairpin RNA and transfection

The mRFP‐GFP‐LC3 plasmid was encapsulated in adenovirus and obtained from Hanbio. The OGG1 short hairpin RNA (shRNA) oligonucleotides and its negative control encapsulated in lentivirus were synthesized by Genechem, and the cells were screened following incubation with 2.5 μg/mL puromycin (631305; TaKaRa) for 10 days. PINK1 siRNA oligonucleotides were synthesized by Sangon. BUMPT cells were transiently transfected with PINK1 siRNA and its negative control constructs using HiPerfect transfection reagent (301705; Qiagen). The sequences of PINK1 siRNA and OGG1 shRNA oligonucleotides were as follows:

PINK1 siRNA, 5′‐GCCTTGGGTTCAGCAAACA;

OGG1 shRNA, 5′‐AAACTTTTTCCGGAATCTGTGG.

### 2.4 Animals and renal IR model

Eight‐ to ten‐week old male C57/BL6 mice were purchased and housed at the Center of Experimental Animals at Wuhan University. Heterozygous OGG1‐targeted allele using CRISPR/Cas9 to remove all exons of the OGG1 gene. C57BL/6J mice with homozygous OGG1‐targeted alleles were generated by interbreeding. All animal protocols were approved by the Animal Care and Use Committee of the Renmin Hospital of Wuhan University.

To generate the mouse renal IR model, all mice (male, 8–10 weeks old, weighing 20–25 g) were first anaesthetised with phenobarbital sodium (60 mg/kg) and then the bilateral renal pedicels were clamped to induce 30 min of ischemia and 24 h of reperfusion. The same procedures were conducted in the sham group without renal pedicle clamping. Th5487 was intraperitoneally injected at 30 mg/kg in each mouse every day for 3 days before the IRI operation, and the mental state of the mice after injection was observed. The last injection was given 2 h before the IR model.

Intercross heterozygous targeted mice were used to generate OGG1 KO homozygous targeted mice. Polymerase chain reaction (PCR) reactions were used to identify homozygous mouse genotypes. Homozygotes are one band with 662 bp. Heterozygotes are two bands with 662 and 834 bp and wild‐type (WT) allele is one band with 834 bp.

PCR Primers 1 (annealing temperature 60.0°C): Product size: 662 bp.

F1: 5′‐GTACATTTTGGAGTCTAAGCACCG‐3′.

R1: 5′‐CAATGTCAAATGTAATGCCAATCCC‐3′.

PCR Primers 2 (Annealing Temperature 60.0°C): Product size: 834 bp.

F1: 5′‐GTACATTTTGGAGTCTAAGCACCG‐3′.

R2: 5′‐ATGTCCCACCTGTAAACTGACTAAT‐3′.

### 2.5 Cell viability assay

BUMPT cells were seeded into 24‐well plates, incubated with the Cell Counting Kit reagent (CCK‐8; Dojindo) for 1 h. Absorbance at 450 nm was measured using a multimode reader (Bio‐Rad).

### 2.6 Renal function analysis

Renal function was evaluated measuring the levels of serum creatinine (SCR; C071; Jilin) and blood urea nitrogen (BUN; C010; Jilin) using commercial kits purchased from Changchun Huili Biotech Corporation. Briefly, whole blood samples were placed at room temperature (15–25°C) for 2 h or at 4°C overnight and then separated in refrigerated centrifuge at 2–8°C for 1000 g/15 min. For SCR measurements, serum samples were added to a pre‐warmed reaction solution for 5 min. The absorbance at 546 nm was recorded at the fifth minute. For BUN measurements, serum samples were mixed with the reaction solution for 1 min. Absorbance at 340 nm was recorded in the second minute. Finally, SCR and BUN levels were calculated based on standard curves.

### 2.7 Renal histology

Kidney tissues were fixed with 4% paraformaldehyde for paraffin embedding and routine analysis. Kidney sections (4 μM) were stained with haematoxylin–eosin. Ten visual fields in the cortico‐medullary region of each section were observed under ×200 microscope. The percentage of damaged renal tubules in the selected fields was plotted to reflect the degree of tubular damage, which was characterized by brush boundary loss, tubule dilatation/flattening, tubule degeneration, tubule casting, vacuolization, and nuclear condensation.

### 2.8 Cell death analysis

Apoptosis was examined in tissues of the renal cortex using the Colorimetric TUNEL Apoptosis Assay Kit (Beyotime; C1098) and co‐stained with lotus tetragonolobus lectin (LTL; Vector) to visualize tubular damage and 4′,6‐diamidino‐2‐phenylindole (DAPI) to display nuclei. For quantification, four fields with 200 cells (recognized using DAPI staining) per field were observed for TUNEL‐positive cells under each experimental condition to evaluate the percentage of apoptosis. Apoptosis was detected by flow cytometry. Cell preparation was conducted using the Annexin V PE Apoptosis kit (559763; BD). In brief, cells were washed twice with cold PBS, resuspended in 1X binding buffer, and analysed using flow cytometry for 1 h.

### 2.9 Mitochondrial isolation

Mitochondria were extracted from cultured proximal tubular cells using a mitochondrial extraction kit (89874; Thermo Fisher Scientific). Briefly, 2 × 10^7^ cells were pelleted by centrifuging the harvested cell suspension in a 2.0 mL microcentrifuge tube at ~850 × *g* for 2 min and then 800 μL of Mitochondria Isolation Reagent A was added. Then, 10 μL of Mitochondria Isolation Reagent B and Reagent C were added successively, mixed and centrifuged at 700 × *g* for 10 min at 4°C. The supernatant was transferred to a new 2.0 mL tube and centrifuged at 12,000 × *g* for 15 min at 4°C. Furthermore, 500 μL of Mitochondria Isolation Reagent C was added to the pellet and centrifuged at 12,000 × *g* for 5 min. Finally, we discarded the supernatant and the pellet containing the isolated mitochondria was mixed with 80 μL RIPA to extract mitochondrial proteins and perform immunoblotting.

### 2.10 Assessment of reactive oxygen species, MMP, superoxide dismutase, adenosine triphosphate content, and the actual concentration of 8‐oxyguanine

Superoxide generation, MMP, and adenosine triphosphate (ATP) content were measured as previously described.^17^ Briefly, reactive oxygen species (ROS) production in BUMPTs was evaluated using dihydroethidium (S0033S; Beyotime) assays. MMP was analysed using JC‐1 staining (C2006; Beyotime). The superoxide dismutase (SOD) content was detected using the Cu/Zn‐SOD and Mn‐SOD Assay Kit with WST‐8 (S0103; Beyotime). ATP generation was measured using an ATP determination kit (C0026; Beyotime). The actual concentration of 8‐oxyguanine was detected using an Elisa Detection kit purchased from Shanghai Jianglai Biotechnology Co., Ltd. (JL45397).

### 2.11 Immunohistochemical and imunofluorescence staining

Paraffin‐embedded renal tissue sections were dewaxed at 65°C, and antigen repair was performed at 95°C in citrate buffer solution (0.01 mol/L, pH 6.0) for 1 h. After being incubated with 5% bovine serum albumin for 1 h, the cells were incubated with primary antibody at 4°C overnight and then fluorescence‐bound secondary antibody (Thermo Fisher Scientific) at 37°C for 2 h in the dark. DAPI (Antgene) was used for nuclear staining. For cell immunofluorescence, cover glass was added to the six‐well plate in advance, and the cells were plated until they grew to an appropriate density. The medium was then removed and the cells were fixed in 4% paraformaldehyde for 30 min. Finally, the subsequent operation is the same as that in tissue immunofluorescence. All images were captured using a fluorescence confocal microscope (Olympus). The fluorescence intensity values were analysed using the ImageJ software developed by National Institutes of Health. The immunohistochemical scoring criteria is the intensity score + the frequency score of positive cells. The intensity was scored as follows: 0, negative; 1, weak; 2, moderate; and 3, strong. The frequency of positive cells was defined as follows: 0, less than 5%; 1, 5%–25%; 2, 26%–50%; 3, 51%–75%: and 4, greater than 75%.

### 2.12 Co‐immunoprecipitation and immunoblot analysis

Briefly, co‐immunoprecipitation was performed using an immunoprecipitation (IP/co‐IP) Kit (Thermo Fisher Scientific). For immunoblotting, total protein samples were separated using sodium dodecyl sulphate–polyacrylamide gel electrophoresis and then transferred to polyvinylidene fluoride membranes (Sigma), which were blocked with protein‐free rapid blocking buffer (Epizyme) for 30 min and incubated overnight at 4°C. HRP‐labelled goat anti‐rabbit/mouse IgG (H + L) antibody (Antgene) was used as the secondary antibody. Finally, the bands were detected by chemiluminescence (Bio‐Rad) and the grey values were analysed using ImageJ software. Statistical analysis was carried out on the ratio of grey value of each group of samples to that of internal parameters, and GraphPad software was used to draw the histogram.

### 2.13 Transmission electron microscopy

Briefly, kidney tissues were dissected from renal ischemia‐reperfused mice and sham‐operated control mice for electron microscopy as previously described.^18^ Kidney tissues were fixed in paraformaldehyde and glutaraldehyde (Sigma‐Aldrich; 340855), post‐fixed in osmium tetroxide (Sigma‐Aldrich; 201030), dehydrated in ethanol, and embedded in Epon (Sigma‐Aldrich; 45345). After polymerization of Epon, blocks were sectioned to generate 70‐nm‐thick sections on a microtome (Leica). The sections were sequentially stained with uranyl acetate (TED PELLA; 19481) and lead citrate (Sigma‐Aldrich; 15326). The stained tissue sections were examined initially at low magnification (×3000) to identify representative proximal tubules. Cells in these tubules were then examined at high magnification (×10,000) to reveal mitochondria. Briefly, digital images with scale bars were collected by transmission electron microscopy (TEM).

### 2.14 Bioinformatics

We downloaded the IR dataset GSE192532 (Data S1) from the GEO dataset of the National Center for Biotechnology Information and screened differentially expressed genes (DEGs) to construct a volcano plot and perform gene ontology (GO) enrichment analysis. Next, VENN analysis was performed using IR datasets GSE192532, GSE71647 (Data S2), and GSE35497 (Data S3) (using OGG1 KO mouse models) to identify the intersection DEGs that represented DEGs related to OGG1 in IRI. Then, the intersection DEGs were imported into STRING for correlation analysis, and Cytoscape software was used to stratify by degree of association. Finally, KEGG and GO enrichment analyses were performed using the Sangerbox. Genes were defined as differential when |logFC| >0.4 and a *p* < 0.05.

### 2.15 Statistical analyses

GraphPad Prism 6 developed by Dr. Harvey Motulsky was used for statistical analysis. All values are expressed as the mean ± SD. Differences between all groups were determined using Student's *t*‐test or one‐way analysis of variance. *p* < 0.05 was regarded as statistically significant difference.

## 3 RESULTS

### 3.1 Expression of OGG1 is upregulated after renal IRI

**FIGURE 1:**
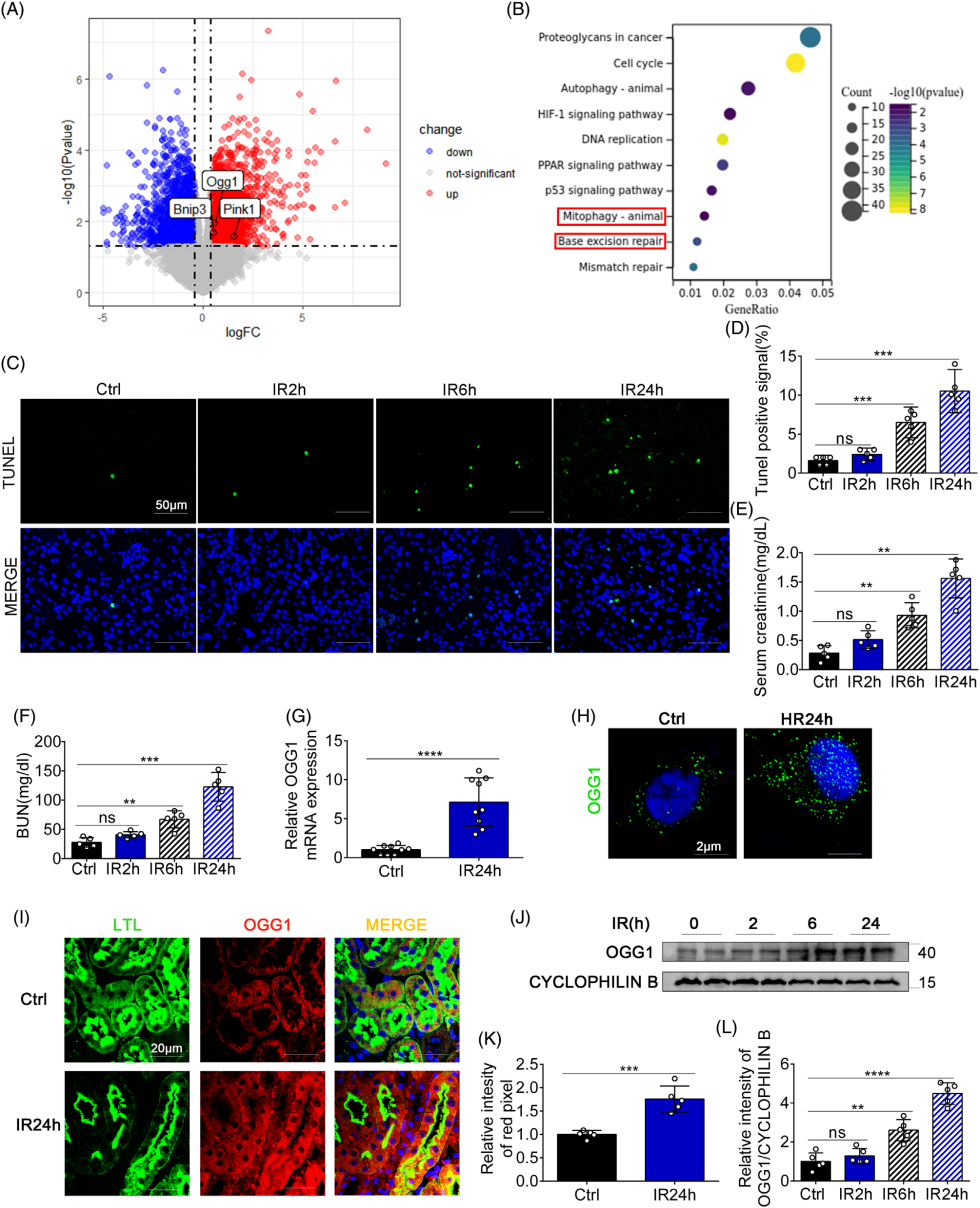
Expression of 8‐oxoguanine DNA glycosylase (OGG1) is upregulated in renal ischemia–reperfusion injury tissue. (A) Volcano plot of the differentially expressed genes (DEGs) based on the GSE192532 dataset (red and green indicate DEGs with a |log2 fold‐change| > 0.4 and false discovery rate < 0.05). (B) Gene ontology enrichment analysis on DEGs. (C–K) 8‐10‐week‐old C57/BL6 mice were treated with renal ischemia for 30 min and reperfusion for 24 h. (C) Representative images of TUNEL staining. (D) The percentage of cells with tunel positive signal was evaluated. (E, F) Blood samples were harvested for measurement of serum creatinine (E) and blood urea nitrogen (F) to indicate renal function decline. (G) OGG1 mRNA expression level was examined by real‐time polymerase chain reaction. (H) Representative confocal microscopy images showing the intracellular localization of OGG1 in Bumpts. (J) Immunoblots of OGG1. (L) Densitometry analysis of OGG1. (I) Representative confocal microscopy images showing the expression of OGG1 in renal cortex and lotus tetragonolobus lectin were used as kidney tubules marker (original magnification, ×600). Scale bar = 20 μm. (K) Quantification of average intensity of red pixel by ImageJ. Data in D–L are expressed as mean ± SD, *n* = 5. *p < 0.05; **p < 0.01; ***p < 0.001; ****p < 0.0001, significant different from the Ctrl group without ischemia–reperfusion treatment. Ctrl, sham control group; IR2h, ischemia for 30 min and reperfusion for 2 h group; IR6h, ischemia for 30 min and reperfusion for 6 h group; IR24h, ischemia for 30 min and reperfusion for 24 h group.

We first examined relevant IR datasets in the GEO database, identified DEGs in IR mice from the GSE192532 dataset, and mapped the volcano plot. Compared with the control, the volcano plot showed an increase in the expression of OGG1 after IR (|logFc| > 0.4, Figure 1A). In addition, DEGs were subjected to GO enrichment analysis, and differences in the biological processes HIF‐1 signalling pathway, mitophagy, and base excision repair were found (Figure 1B). We established bilateral renal IRI in C57BL/6 mice as previously described. At reperfusion time of 24 h, SCR and BUN were upregulated, indicating successful modelling (Figure 1E,F). An increase in TUNEL staining of apoptotic cells over time after renal IRI indicated the occurrence of cell death (Figure 1C,D). RNAs was collected and analysed using RT‐PCR, and the results showed that the expression of OGG1 mRNA increased following renal IR treatment (Figure 1G). Immunofluorescence staining of OGG1 in BUMPTs showed the expression of OGG1 was increased in cytosol and nucleus during renal IRI (Figure 1H). Consistently, immunoblot analysis showed that the protein levels of OGG1 (Figure 1J,L) were increased. Immunofluorescence staining of C57BL/6 mice kidney tissues showed that the fluorescence intensity levels of OGG1 (red signal) were increased in epithelial tubular cells during renal IRI (Figure 1I,K). Together, these findings demonstrate that OGG1 expression is enhanced in response to IRI.

### 3.2 OGG1‐KO ameliorates renal IRI

**FIGURE 2:**
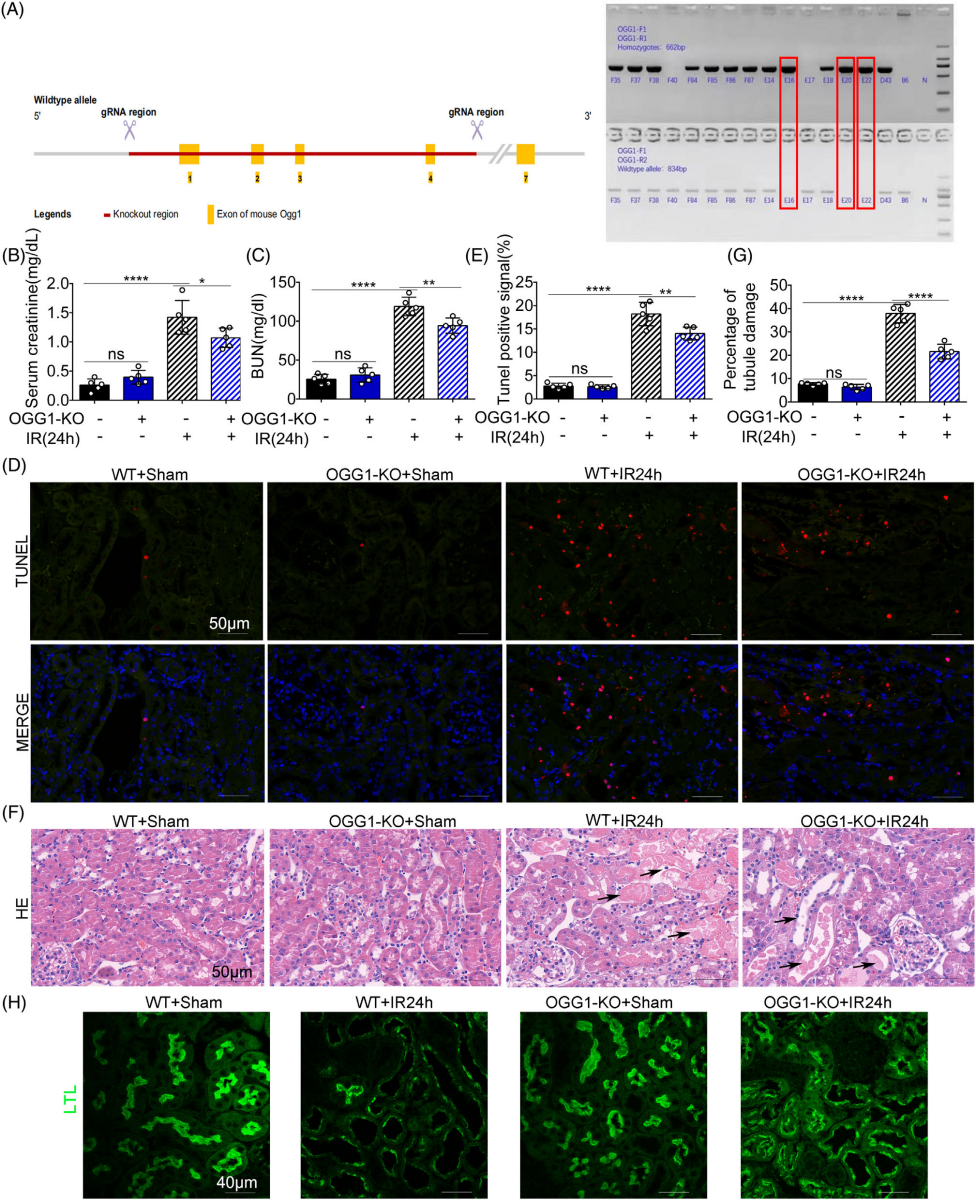
8‐Oxoguanine DNA glycosylase (OGG1) knockout (KO) ameliorates renal ischemia–reperfusion injury. OGG1 KO and wild‐type (WT) mice were treated with renal ischemia for 30 min and reperfusion for 24 h. (A) The knockout strategy of OGG1 gene and genotypes of OGG1 KO mice were identified by polymerase chain reaction. (B, C) Blood samples were harvested for measurement of serum creatinine (B) and blood urea nitrogen (C) to indicate renal function decline. (D) Representative images of TUNEL staining. (E) The percentage of cells with tunel positive signal was evaluated. (F) Representative images of haematoxylin–eosin staining. The arrows indicated tubule dilatation, tubule degeneration, tubule casting, vacuolization, and nuclear condensation. (G) Quantification analysis of tubular damage by counting the renal tubules with signs of injury. (H) Representative images showing the extent of damage to the brush border of the renal tubules, and lotus tetragonolobus lectin was used as kidney tubules marker (original magnification, ×400). Scale bar = 40 μm. Mean ± SD, *n* = 5. *p < 0.05; **p < 0.01; ****p < 0.0001 compared with WT‐Sham group or OGG1 KO + IR24h group. IR24h, ischemia for 30 min and reperfusion for 24 h group; OGG1‐KO, OGG1 knockout group. WT + Sham, wild‐type sham group; OGG1‐KO + Sham, OGG1 knockout sham group; WT + IR24h, wild‐type group with ischemia for 30 min and reperfusion for 24 h; OGG1‐KO + IR24h, OGG1 knockout group with ischemia for 30 min and reperfusion for 24 h; Th5487 + IR24h, Th5487 treatment group with ischemia 30 min and reperfusion for 24 h.

A schematic of the strategy for the generation of OGG1 KO mice using the CRISPR/Cas9 method is shown in Figure 2A. The KO mice were further confirmed by RT‐PCR using mouse tail DNA (Figure 2A). OGG1‐KO mice exhibited a normal morphology and function (Figure 2B,F). After IRI treatment, the OGG1‐KO + IR24h (ischemia for 30 min and reperfusion for 24 h) group showed lower SCR and BUN levels than the WT + IR24h group (Figure 2B,C). Consistently, TUNEL staining showed lower cell death in the OGG1‐KO group than in WT mice (Figure 2D,E). HE staining results showed that IRI induced necrosis, dilation and vacuolar degeneration of renal tubules in the WT + IR24h group, which were ameliorated in the OGG1‐KO mice (Figure 2F,G). LTL was used as a fluorescent dye to label the brush border of renal tubules. IRI led to diminished LTL fluorescence in OGG1‐WT mice, which was partially preserved in OGG1‐KO mice (Figure 2H). These results suggest that OGG1‐KO alleviates acute tubular injury following IRI.

### 3.3 OGG1 inhibitor Th5487 ameliorates renal IRI

**FIGURE 3:**
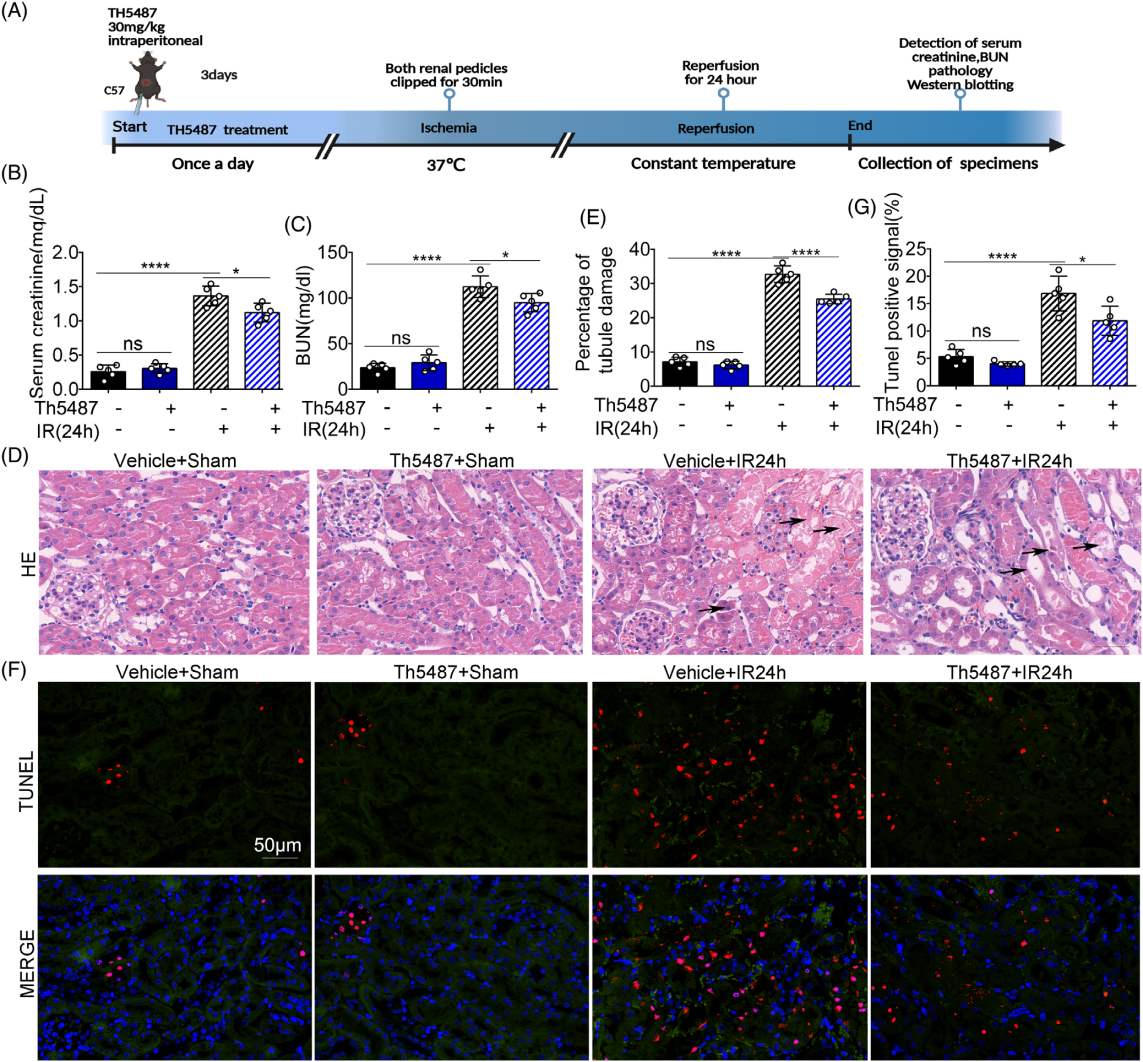
8‐Oxoguanine DNA glycosylase specific inhibitor Th5487 ameliorates renal ischemia–reperfusion injury. C57BL/6 mice were treated with renal ischemia for 30 min and reperfusion for 24 h. Th5487 (30mg/kg) was intraperitoneally injected into each mouse every day for 3 days before ischemia–reperfusion operation. (A) Modelling of ischemia–reperfusion injury in Th5487 treatment mice and vehicle treatment mice. (B) Serum creatinine measurement, (C) blood urea nitrogen measurement. (D) Representative images of haematoxylin–eosin staining. The arrows indicated tubule dilatation, tubule degeneration, tubule casting, vacuolization, and nuclear condensation. (E) Quantification analysis of tubular damage by counting the renal tubules with signs of injury. (F) Representative images of TUNEL staining. (G) The percentage of TUNEL positive cells. Mean ± SD, *n* = 5. ****p < 0.0001 compared with Vehicle + Sham group, *p < 0.05; ****p < 0.0001 compared with Th5487 + IR24h group. IR24h, ischemia for 30 min and reperfusion for 24 h group; Th5487, Th5487 treatment group; Vehicle + Sham, vehicle treatment with sham group; Th5487 + Sham, Th5487 treatment with sham group; Vehicle + IR24h, vehicle treatment group with ischemia for 30 min and reperfusion for 24 h; Th5487 + IR24h, Th5487 treatment group with ischamia for 30 min and reperfusion for 24 h.

We investigated the effect of the OGG1 inhibitor Th5487 in renal IRI. As shown in Figure 3A, Th5487 (30 mg/kg) was intraperitoneally injected into each mouse every day for 3 days before the IR operation. Mice in the Th5487 + IR24h group had normal renal function after sham surgery but lower levels of SCR and BUN after IRI compared to those in the vehicle group (Figure 3B,C). Furthermore, the Th5487 treatment group showed partial tubular dilation and vacuolar degeneration after IR24h, with a reduced tubular injury score. In comparison, nuclear pyknosis and necrosis of renal tubules were observed in the vehicle group after IR24h (Figure 3D,E). TUNEL staining showed that the Th5487 + IR24h group had lower apoptosis rates (Figure 3F,G).

### 3.4 OGG1‐knockdown suppresses BUMPT apoptosis after HR

**FIGURE 4:**
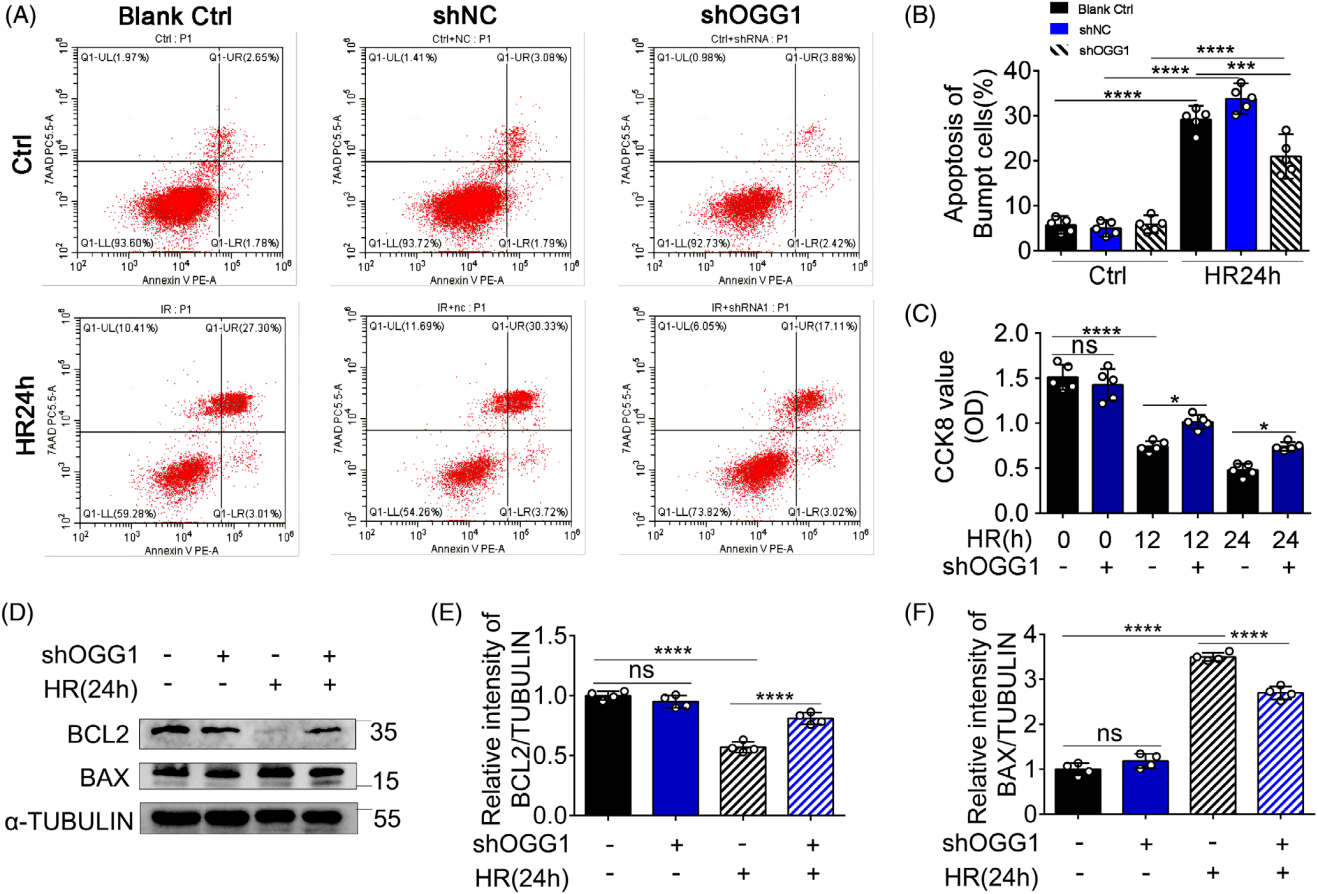
8‐Oxoguanine DNA glycosylase (OGG1) knockdown suppresses BUMPT cells apoptosis after HR24h. BUMPT cells were transfected with NC shRNA and OGG1 shRNA. OGG1 knockdown BUMPT cell lines were obtained by transfection of OGG1 lentivirus and screening for 10 days with puromycin. After transfection, cells were hypoxia for 24 h and reoxygenation for 4 h. (A) Flow cytometry following PE and 7AAD staining. (B) Percentage of apoptosis. The percentage of late apoptotic and early apoptotic cells in total cells was set as apoptotic positive rate. (C) Cell viability assay evaluated by Cell Counting Kit detection. (D) The whole‐cell lysate was collected for immunoblot analysis of BCL2, BAX, and α‐tubulin. (E, F, H, I) Densitometry of BCL2 and BAX. Mean ± SD, *n* = 5. ****p < 0.0001 compared with Ctrl group or shOGG1 + HR24h group. Ctrl, control group; HR24h, hypoxia for 24 h and reoxygenation for 4 h group; Blank Ctrl, bumpt cells normal control groups; shNC, negative control group transfected with shRNA; shOGG1, OGG1 knockdown group transfected with shRNA.

Next, we evaluated the effect of OGG1‐knockdown on BUMPT apoptosis in response to hypoxia for 24 h and reoxygenation for 4 h (HR24h). Flow cytometry analysis of BUMPTs following PE and 7AAD staining showed increased tubular apoptosis after HR24h, which was suppressed by OGG1‐knockdown (Figure 4A,B). Consistently, the cell viability, measured by CCK‐8 assay, decreased significantly after HR24h treatment (Figure 4C). In contrast, the OGG1‐knockdown group transfected with shRNA and subjected to HR24h (shOGG1 + HR24h group) had higher levels of viability than the negative control group transfected with shRNA and subjected to HR24h (shNC + HR24h group). Immunoblot analysis showed that the expression levels of Bax increased and those of Bcl2 decreased after HR24h (Figure 4D–F). Furthermore, the shOGG1 + HR24h group had higher levels of Bcl2 and lower levels of Bax than the shNC + HR24h group. These data indicate that knockdown of OGG1 reduces renal tubular cell death after HR24h.

### 3.5 OGG1‐knockdown alleviates BUMPT mitochondrial function after HR

**FIGURE 5:**
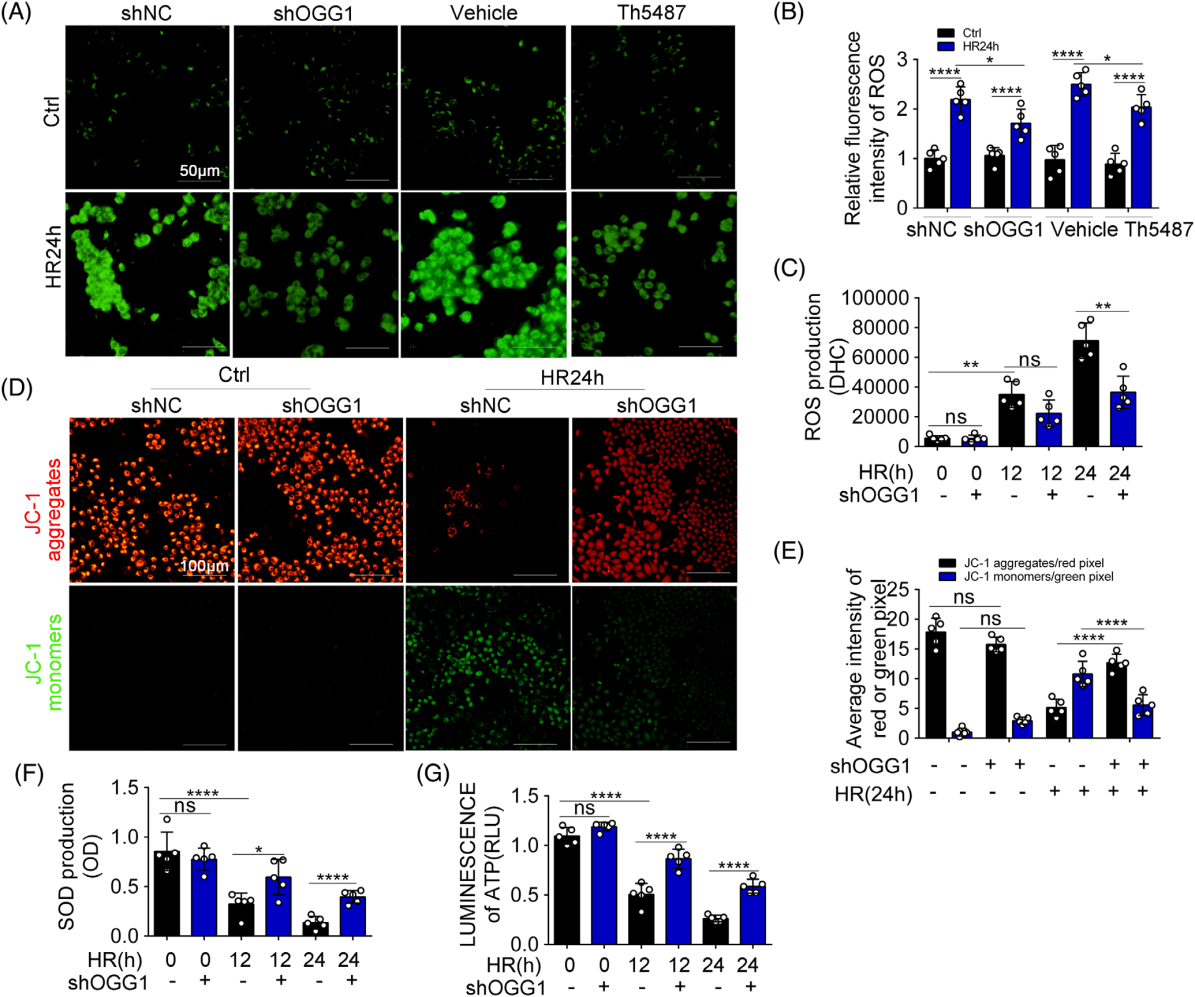
8‐Oxoguanine DNA glycosylase (OGG1) knockdown alleviates BUMPTs mitochondrial function after HR24h. (A) Representative DCFDA staining image of BUMPTs from each group (original magnification, ×400), scale bar = 50 μm, *n* = 5. (B) Quantitative relative fluorescence intensity analysis of reactive oxygen species (ROS) of (A). (C, F) Quantitative analysis of ROS and superoxide dismutase content by fluorescence spectrophotometer, *n* = 5. (D) JC‐1 staining to assess membrane potential (original magnification, ×200), scale bar = 100 μm, *n* = 5. (E) Quantitative analysis of membrane potential (red indicates JC‐1 aggregates and green indicates JC‐1 monomers). (G) Chemiluminescence method to detect ATP production, *n* = 5. Mean ± SD. **p < 0.01; ***p < 0.001; ****p < 0.0001 compared with shNC or Vehicle group. *p < 0.05; **p < 0.01; ****p < 0.0001 compared with shOGG1 + HR24h group. Ctrl, control group; HR24h, hypoxia for 24 h and reoxygenation for 4 h group; shNC, negative control group transfected with shRNA; shOGG1, OGG1 knockdown group transfected with shRNA; Vehicle, vehicle group; Vehicle + IR24h, vehicle treatment group with hypoxia for 24 h and reoxygenation for 4 h.

Mitochondrial dysfunction, including MMP collapse and mitochondrial permeability transition pore opening, are crucial factors contributing to IRI‐related damage that can result in cell death. MMP was measured using the probe JC‐1. When MMP depolarizes, JC‐1 exists as a monomer and emits green fluorescence. The results showed that MMP decreased, and apoptosis occurred after HR24h (Figure 5D,E). In addition, HR24h induced lower rates of apoptosis in the shOGG1 groups than in the shNC + HR24h groups. HR24h treatment increased redox status imbalance with increased ROS and decreased SOD (Figure 5A–C,F). ROS and SOD levels were partially normalized in the shOGG1 + HR24h groups (Figure 5C,D). We used a chemiluminescence method to determine ATP production and found a reduction in ATP production over time in cells subjected to HR24h treatment, which was preserved in OGG1‐knockdown cells(Figure 5G). Together, these results indicate that OGG1 aggravates mitochondrial dysfunction in BUMPTs during HR.

### 3.6 OGG1 interacts and inhibits PINK1 in BUMPTs

**FIGURE 6:**
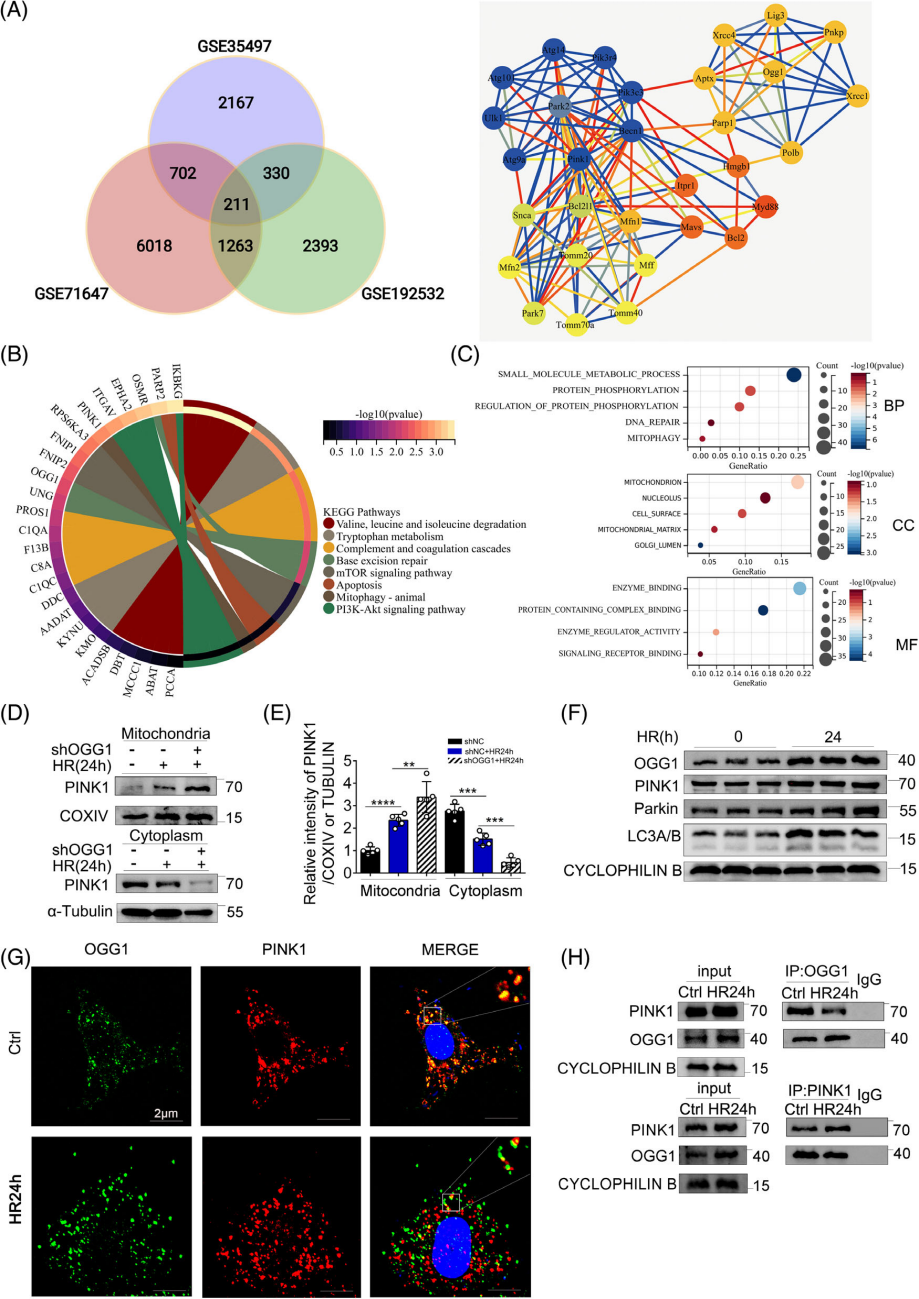
8‐Oxoguanine DNA glycosylase (OGG1) inhibited PINK1 by interaction with PINK1 in BUMPTs. (A–C) Bioinformatics analysis. (A) VENN analysis was performed using ischemia–reperfusion datasets GSE192532, GSE71647, and GSE35497 (using OGG1 knockout mouse models) to identify the intersection differentially expressed genes (DEGs) that represented DEGs related to OGG1 in ischemia–reperfusion injury. OGG1 and PINK1 were placed on string website to search for association, and then Cytoscape software was imported to generate protein interaction diagrams according to degree of correlation. (B, C) The 211 intersection DEGs were imported into the Sangerbox website for KEGG pathway enrichment analysis (B) and gene ontology function enrichment analysis (C). (F) Immunoblots of OGG1, PINK1, Parkin, and LC3A/B in BUMPTs. (G) Double immunofluorescence staining of OGG1 and PINK1 in different groups (original magnification, ×1000). Scale bar = 2 μm. (H) Representative co‐immunoprecipitation results of the interaction between PINK1 and OGG1 in BUMPTs hypoxia for 24 h and reoxygenation for 4 h. (D, E) Immunoblot analysis of PINK1 accumulation in mitochondria and cytoplasm and evaluation of the effects of OGG1 knockdown. BUMPT cells transfected with shRNA for OGG1 konckdown and negative control were subjected to HR24h. These cells were harvested and fractionated to cytoplasm and mitochondria for immunoblot analysis of PINK1, COXIV (mitochondrial marker), and α‐tubulin (cytoplasm marker). (D) Representative blots. (E) Densitometry of PINK1. Mean ± SD. **p < 0.01; ****p < 0.0001 compared with shNC group or shOGG1 + HR24h group in mitochondria. ***p < 0.001 compared with shNC group and shOGG1 + HR24h group in cytoplasm. Ctrl, control group; HR24h, hypoxia for 24 h and reoxygenation for 4 h group; shOGG1, OGG1 knockdown group transfected with shRNA.

To further explore the protective mechanism of OGG1‐KO on renal IR, we downloaded the IR datasets (GSE192532 and GSE71647) and the OGG1 KO dataset (GSE35497) from the GEO database to perform VENN analysis and identify the intersection DEGs. The WENN analysis, described above, revealed 211 intersection DEGs between the three datasets that contained OGG1 and PINK1 (Figure 6A). We then introduced these 211 DEGs to the Sangerbox website and performed KEGG pathway and GO enrichment analyses. KEGG results showed that the base excision repair and mitophagy pathways were enriched (Figure 6B). The GO results were divided into three sections: biological processes, cellular components, and molecular functions. The biological processes mitophagy and DNA repair, the cellular component mitochondrion, and the molecular function enzyme binding suggested a relationship between OGG1 and mitophagy (Figure 6C). The upregulation of OGG1 and the mitophagy pathway in BUMPT cells with IR was verified using immunoblotting (Figure 6F). Next, we explored the interaction between OGG1 and PINK1, which was verified by co‐immunoprecipitation (co‐IP). Co‐immunoprecipitation of OGG1 and PINK1 showed that OGG1 and PINK1 could bind to each other, and the binding decreased after HR24h (Figure 6H). Consistently, immunofluorescence colocalization analysis showed that OGG1 and PINK1 were partially colocalized under normal conditions, but colocalization was reduced under HR24h conditions (Figure 6G). To further explore whether OGG1 could affect PINK1 expression in mitochondria, we examined PINK1 expression levels in isolated mitochondria and cytoplasm using immunoblot. Interestingly, that PINK1 expression was increased in the mitochondria of the shNC + HR24h group, and PINK1 was further increased after OGG1 knockdown (Figure 6D,E). Together, these results indicate that OGG1 interacts with PINK1 and OGG1 represses PINK1 translocation in the mitochondria.

### 3.7 PINK1 is an essential protein for mitophagy in cultured proximal tubular cells

**FIGURE 7:**
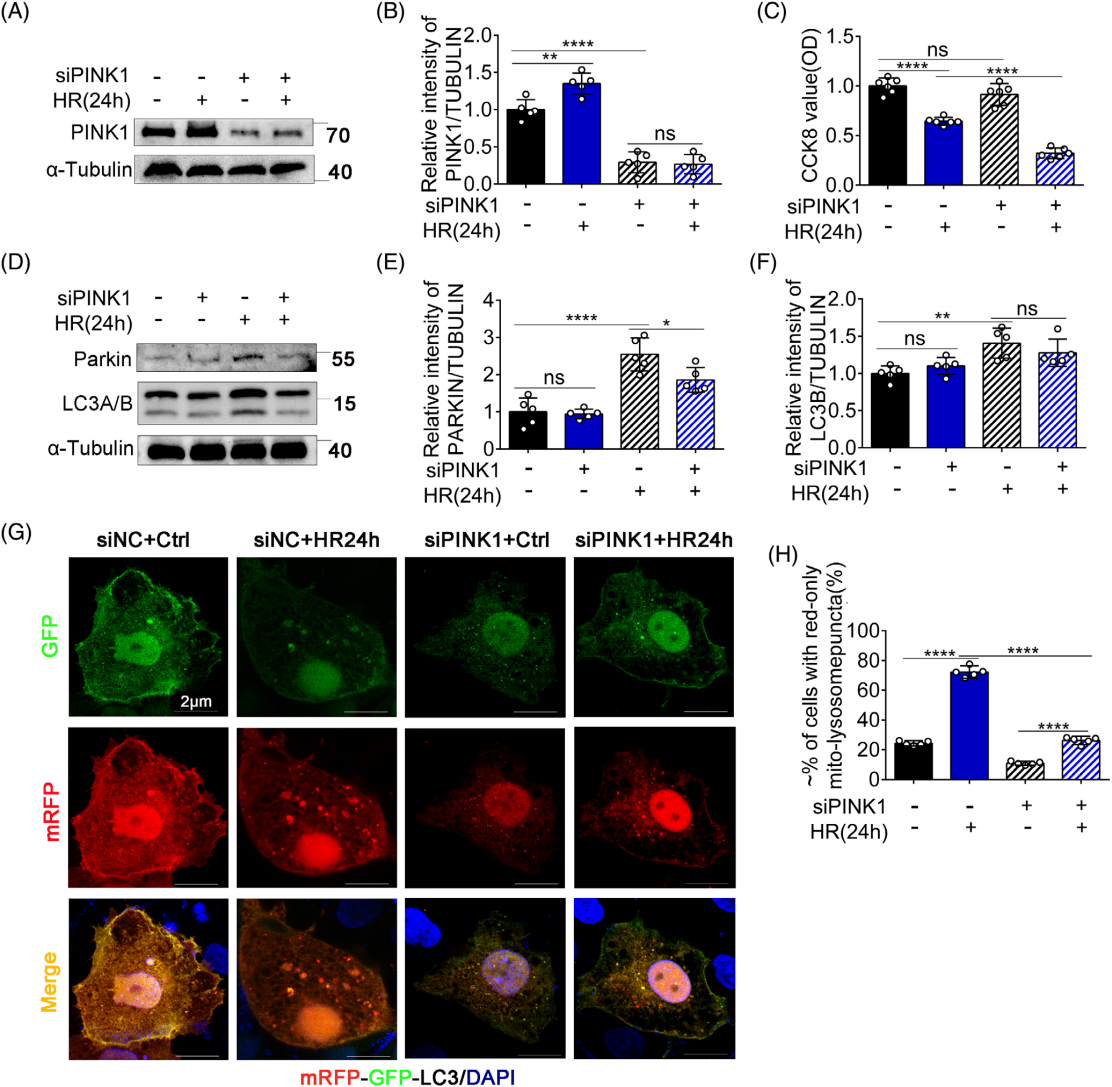
PINK1 is an essential protein for mitophagy in cultured proximal tubular cells. (A–F) siNC cells and PINK‐siRNA cells were hypoxia for 24 h and reoxygenation for 4 h. Cell viability was detected and we collected whole‐cell lysate for immunoblot analysis of PINK1, Parkin, LC3A/B, and α‐tubulin. (A, D) Representative blots. (B, E, F) Densitometry analysis of PINK1, Parkin, and LC3A/B. BUMPT cells were transfected with PINK1 siRNA. At 48 h after transfection, the cells were treated with HR24h to collect the whole cells lysate for immunoblot analysis. (G, H) Inhibition of mitophagosome formation after HR24h by silencing PINK1. The BUMPTs were transfected with PINK1 siRNA or negative control siRNA and 24 h later these cells were transfected with mRFP‐GFP‐LC3. After hypoxia for 24 h and reoxygenation for 4 h, the cells were stained with DAPI (blue) observed from confocal microscopy. (G) Representative images. (H) Quantification of cells with red‐only puncta. Mean ± SD, *n* = 5. **p < 0.01; ****p < 0.0001 compared with Ctrl group or PINK1‐siRNA + HR group. siPINK1, PINK1 knockdown group transfected with siRNA; HR24h, hypoxia for 24 h and reoxygenation for 4 h group; siNC + Ctrl, negative control group transfected with siRNA; siNC + HR24h, negative control group transfected with siRNA and hypoxia for 24 h and reoxygenation for 4 h; siPINK1 + Ctrl, PINK1 knockdown control group; siPINK1 + HR24h, PINK1 knockdown group transfected with siRNA and hypoxia for 24 h and reoxygenation for 4 h.

Next, we examined the function of PINK1 in HR‐induced mitophagy. PINK1 was knocked down using siRNA, and the knockdown efficiency was verified using immunoblotting (Figure 7A,B). As shown in Figure 7D–F, Parkin and LC3 were not activated in the PINK1‐knockdown (siPINK1) + HR24h group, suggesting that the PINK1 mitophagy pathway was inhibited. In addition, we examined the effect of PINK1‐knockdown on the delivery of mitochondria to lysosomes in mRFP‐GFP‐LC3 (a tandem fluorescent‐tagged mitochondrial targeting sequence of the mitophagy protein LC3)‐expressing cells. As shown in Figure 7G,H, control cells displayed yellow staining of tubular or filamentous mitochondria with merging of green (GFP) and red (mRFP) signals. HR elevated the number of mito‐lysosome puncta; however, this number was not significant in the siPINK1 group. Moreover, the CCK‐8 assay demonstrated that the siPINK1 + HR24h group had a lower cell viability than the other groups (Figure 7C). Taken together, these results indicate that PINK1‐regulated mitophagy to reduce HR damage.

### 3.8 OGG1 inhibits mitophagy by interacting PINK1 during HR in BUMPTs

**FIGURE 8:**
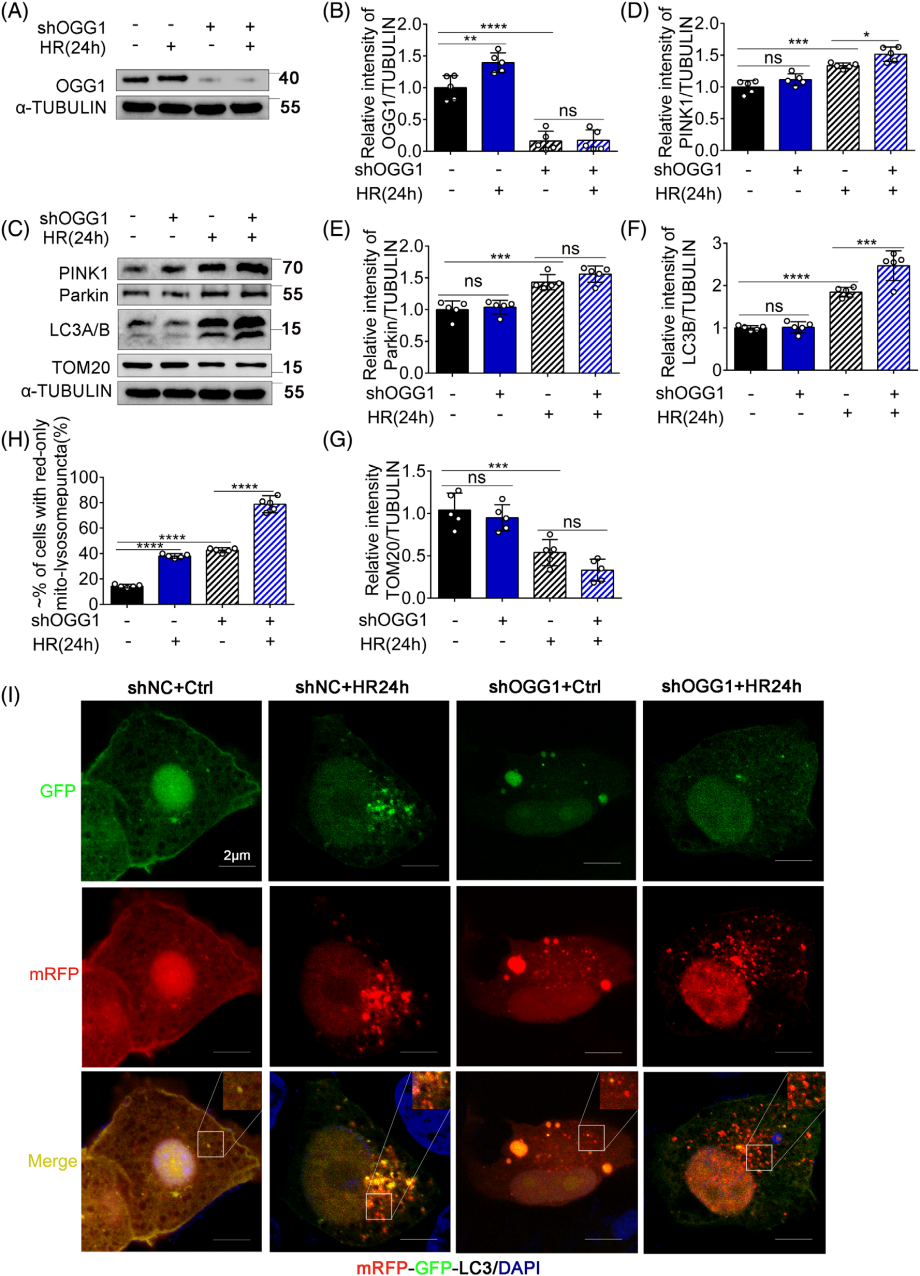
8‐Oxoguanine DNA glycosylase (OGG1) inhibited mitophagy by interacting PINK1 during hypoxia‐reoxygenation in BUMPTs. (A–G) siNC cells and shOGG1 cells were treated with hypoxia for 24 h and reoxygenation for 4 h. The whole‐cell lysate was collected for immunoblot analysis of OGG1, PINK1, Parkin, LC3A/B, Tom20, and α‐tubulin. (A, C) Representative immunoblots. (B, D, E–G) Densitometry analysis of OGG1, PINK1, Parkin, LC3A/B, Tom20, and α‐tubulin. BUMPT cells were transfected with OGG1 shRNA. After transfection, the cells were treated with HR24h to collect the whole cells lysate for immunoblot analysis. (G, H) Promotion formation of mitophagosome after HR24h by silencing OGG1. The BUMPTs were transfected with OGG1 shRNA or negative control shRNA and then they were transfected with mRFP‐GFP‐LC3. After hypoxia for 24 h and reoxygenation for 4 h. Finally, the cells were stained with DAPI (blue) observed from confocal microscopy. (G) Representative images. (H) Quantification of cells with red‐only puncta. Mean ± SD, *n* = 5. *p < 0.05; **p < 0.01; ***p < 0.001; ****p < 0.0001 compared with shNC + Ctrl group or shOGG1 + HR24h group. shOGG1, OGG1 knockdown group transfected with shRNA; HR24h, hypoxia for 24 h and reoxygenation for 4 h group; shNC + Ctrl, negative control group transfected with shRNA; shNC + HR24h, negative control group transfected with shRNA and hypoxia for 24 h and reoxygenation for 4 h; shOGG1 + Ctrl, OGG1 knockdown control group; shOGG1 + HR24h, OGG1 knockdown group transfected with shRNA and hypoxia for 24 h and reoxygenation for 4 h.

We continued to explore the regulatory function of OGG1 on the PINK1 mitophagy pathway in BUMPTs. As shown in Figure 8A,B, OGG1 was efficiently knockdown using lentivirus containing OGG1 shRNA. Interestingly, knockdown of OGG1 increased the expression of PINK1, Parkin, and LC3B and decreased the expression of TOM20 in BUMPTs subjected to HR24h compared to other groups, indicating that OGG1 represses mitophagy (Figure 8C–G). In this setting, we further examined the effect of OGG1 knockdown on autophagic activity in mRFP‐GFP‐LC3 expressing cells. As shown in Figure 8H,I, the shNC group displayed yellow staining of tubular or filamentous mitochondria with merging of green (GFP) and red (mRFP) signals. In sharp contrast, the number of red puncta increased in HR24h cells, indicating an increase in autolysosome formation. In the shOGG1 + HR24h group, the number of red puncta increased and exceeded that of the yellow puncta, indicating that autophagic activity was higher than in the other groups. Together, these findings suggest that mitophagy is further activated in OGG1‐knockdown BUMPTs subjected to HR24h.

### 3.9 OGG1‐KO further activates mitophagy pathway in mice treated with IRI

**FIGURE 9:**
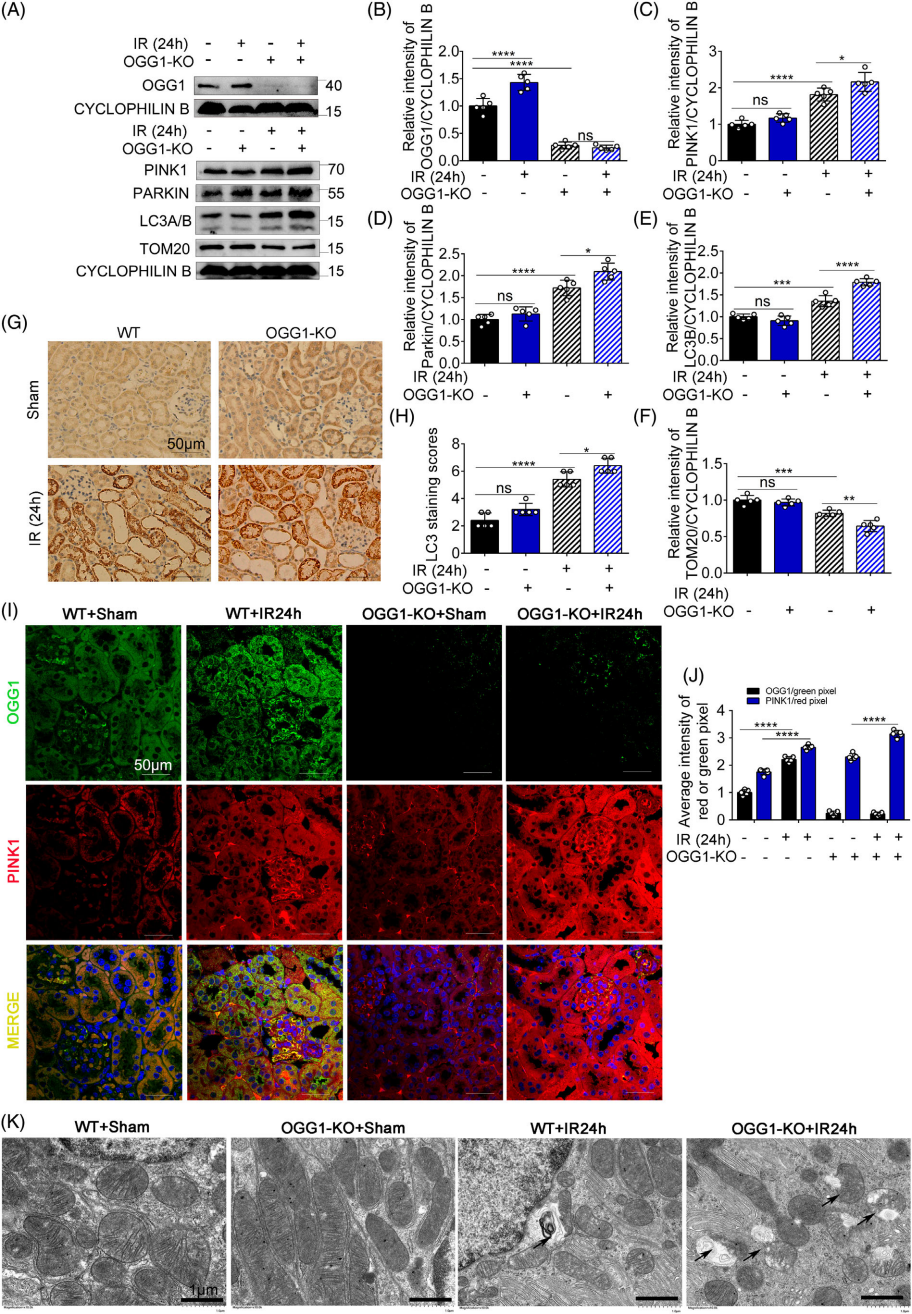
8‐Oxoguanine DNA glycosylase (OGG1) knockout (KO) further activated mitophagy pathway in mice treated with ischemia–reperfusion. (A–F) OGG1‐KO and wild‐type (WT) mice were treated with ischemia for 30 min and reperfusion for 24 h. (A) Kidney cortex tissues were gathered from OGG1‐KO and WT mice for immunoblot analysis of OGG1, PINK1, Parkin, LC3A/B, Tom20, and Cyclophilin B. (B–F) Densitometry of OGG1, PINK1, Parkin, LC3B, and Tom20. (G) Representative of immunohistochemistry staining of renal tubular LC3 in each group. (H) Quantification of immunohistochemistry staining of tubular LC3 in each group. (I) Double immunofluorescence staining of renal tubular OGG1 and PINK1 in the different groups (original magnification, ×400). (J) Quantitative analysis of the average intensity of the red or green pixel (red indicates PINK1 and green indicates OGG1). Scale bar = 50 μm. (K) Representative TEM images of mitochondrial morphology in proximal tubular cells. Scale bar = 1 μm (the arrows indicated mitophagy and mitochondria‐derived vesicles appeared). Mean ± SD, *n* = 5. *p < 0.05; **p < 0.01; ***p < 0.001; ****p < 0.0001 compared with WT + Sham group or OGG1‐KO + IR24h group. IR24h, ischemia for 30 min and reperfusion for 24 h group; OGG1‐KO, OGG1 knockout group. WT + Sham, wild‐type sham group; OGG1‐KO + Sham, OGG1 knockout sham group; WT + IR24h, wild‐type group with ischemia for 30 min and reperfusion for 24 h; OGG1‐KO + IR24h, OGG1 knockout group with ischamia for 30 min and reperfusion for 24 h.

We investigated the role of OGG1 in regulating the PINK1/Parkin mitophagy pathway in renal IRI in vivo. As shown in Figure 9A,B, the OGG1‐KO was confirmed at the protein level. OGG1‐KO mice had high protein expression levels of PINK1, Parkin, and LC3B and low protein expression levels of TOM20. The expression levels of PINK1, Parkin, and LC3B were further elevated and those of TOM20 were further reduced in the OGG1‐KO + IR24h group (Figure 9C–F). These results suggest the involvement of OGG1 in mitophagy in an IRI mouse model. In addition, the immunohistochemical staining results for LC3 showed further accumulation of LC3 in the OGG1‐KO group (Figure 9G,H). The tissue fluorescence double staining results of OGG1 and PINK1 also demonstrated that the expression levels of PINK1 were increased in OGG1‐KO mice and the tissue damage was improved after OGG1‐KO, which was consistent with previous results (Figure 9I,J). The TEM results in proximal tubular cells demonstrated that mitophagy level increased in OGG1‐KO + IR24h group compapred to the WT + IR24h group (Figure 9K). Together, OGG1 could impair mitophagy by inhibiting PINK1 activity in renal IRI.

## 4 DISCUSSION

The oxidative stress response during IR is a key pathogenetic mechanism in renal IRI^19^ and mitophagy activation helps maintain mitochondrial quality control and preserve mitochondrial function in renal IRI^20^, ^21^, ^22^ by preventing ROS production and apoptotic factor release from mitochondria.^23^ OGG‐1, an enzyme that repairs oxidative DNA damage, specifically binds to 8‐oxoguanine in DNA duplexes and participates in base excision repair. Some studies have found that the expression of OGG1 is upregulated in IRI models of the brain^9^ and kidney,^10^ but the role of OGG1 in IRI has rarely been studied. In recent years, OGG1 was found to activate inflammatory pathways in response to oxidative stress.^24^ The function of the protein interaction and transcriptional regulation of OGG1 came into view beyond base excision repair. Our current study is the first to evaluate its role in a renal IRI model, demonstrating that OGG1 is activated in ischemic renal injury and represses mitophagy by interacting with PINK1 and inhibiting its translocation to mitochondria. Our results demonstrated that OGG1 inhibition or gene KO alleviated renal IRI, and that OGG1 deficiency activated mitophagy, a critical process in mitochondrial quality control, by promoting PINK1 mitochondrial translocation.

Studies on the role of OGG1 in IRI in various organs are limited. Lan et al.^25^ found that the expression levels of OGG1 increases after ischemia, and the activation of OGG1 constitutes a significant endogenous mechanism that protects the brain against oxidative neuronal injury caused by ischemia. However, it has also been demonstrated that OGG1‐KO mice presented decreased mtDNA damage after 60 min of myocardial IR, and the integrity and levels of mtDNA were restored after 6 weeks of reperfusion without changes in cardiac function, indicating that OGG1‐mediated mtDNA repair may not be important for the maintenance of cardiac function during IRI in vivo.^26^ The heart and kidneys consume the most oxygen and have the most mitochondria per milligramme of tissue compared to other visceral organ, leading to susceptibility to oxidative stress.^27^ In renal IRI, OGG1 mRNA levels in the cortex and outer medulla were found to increase after 1–7 days of renal IR.^10^ In our study, we found increased OGG1 expression in proximal tubular cells subjected to HR24h and in the renal cortex under IRI, suggesting that OGG1 might play a role in the regulation of renal IRI. Th5487, a specific active‐site inhibitor of OGG1, was confirmed to be well tolerated in mice.^24^ Mice treated with Th5487 and OGG1 KO mice were resistant to renal IRI, indicating that OGG1 contributes to tubular injury during renal IRI.

OGG1 is a DNA glycosylase enzyme with apurinic/apyrimidinic (AP) site lytic activity that removes ROS‐induced 8‐oxoG.^28^ However, under acute ROS stimulation, the cysteine residues of OGG1 are oxidized and inactivated, and the repair function of OGG1 is almost completely lost. Repair enzyme activity can be restored when the redox level is restored.^29^ The most significant difference between OGG1 and other subfamilies such as OGG2 and AGOG is the additional N‐terminal A domain, which mediates OGG1 protein–protein interactions and does not undergo oxidative modification.^30^ The human OGG1α isoform is expressed in the cytoplasm, nucleus, and mitochondria, whereas the human OGG1β isoform is only expressed in the mitochondria. The absence of mitochondrial translocation signals in the A domain of hOGG1 N‐terminal seems to prevent its localization to mitochondria and supports the role of the N‐terminal A domain in protein localization^31^, ^32^, ^33^ the N‐terminal domain appears to be an important structure through which OGG1 interacts with related proteins and localizes in mitochondria.

In addition to its function in DNA damage repair, recent research has shown that OGG1 can interact with certain proteins to regulate inflammation, fibrosis and autophagy. For example, OGG1 inhibitors induce type I IFN release via the mtDNA‐cGAS‐Sting‐IRF4‐IFN‐β axis, thereby reducing progression of inflammation.^34^ OGG1‐deficient mice have good survival capacity and show significant resistance to acute and systemic inflammation.^35^ Wang observed that OGG1 promoted TGF‐β1‐induced cell transformation and activated Smad2/3 by interacting with Smad7 and that the interaction between OGG1 and the TGF‐β/Smad axis modulates the cell transformation process in fibroblasts. In addition, they demonstrated that OGG1 deficiency relieved pulmonary fibrosis and decreased the expression of Smad7 and phosphorylation of SMAD2/3 in bleomycin‐treated mice.^36^ We found that as reperfusion time increased, OGG1 expression increased continuously, whereas the base repair function of OGG1 did not show a corresponding increase, which indicated that there might be other biological functions of OGG1 in renal IRI. Bax inhibitor‐1 delivers anti‐apoptotic signals for mitochondria, which can reduce renal failure, inflammation and tubule death in acute kidney injury (AKI) and protect renal function.^37^ We found that the decrease of Bax expression in OGG1 KO mice in renal IRI activated the anti‐apoptotic process and helpful to preservation of mitochondrial integrity compared to the control IR group.

DNA damage and repair are closely associated with autophagy.^38^ OGG1 binds to the promoter of the autophagy molecule Atg7 and regulates its activity.^15^ In addition, OGG1 negatively regulates the release of inflammatory cytokines through molecular protein interactions that coordinate autophagy pathways in hyperoxygen‐induced lung injury.^39^ Hooten et al.^40^ found that OGG1 binds directly to poly(ADP‐ribose) polymerase 1 (PARP‐1) through its N‐terminal region, and this interaction is enhanced by oxidative stress. ROS‐induced DNA damage and PARP‐1 are required for optimal induction of starvation‐induced autophagy.^41^ In addition, PARP1 can induce ATP depletion and suppress the mTOR pathway to regulate autophagy initiation.^42^ Under oxidative stress, OGG1 may interact with PARP1 to regulate autophagy. Another study found^16^ that OGG1 was activated by the autophagy inhibitor bafilomycin and in an autophagy‐deficient Atg5‐KO mouse cell model under nutrient deprivation. Nevertheless, the pharmacological activation of autophagy did not induce OGG1 loss. Our study found that OGG1‐KO activated the PINK1–Parkin mitophagy pathway in renal IRI, indicating that there may be a feedback mechanism between mitophagy and OGG1 activation. In addition, we demonstrated that the KO of OGG1 improved hypoxic‐reoxygenation‐induced apoptosis, kidney tissue damage, and kidney dysfunction in vitro and in vivo. The PINK1–Parkin pathway has been implicated in the regulation of mitophagy‐independent quality control mechanisms involving mitochondrial‐derived vesicles, which selectively transport cargo from the mitochondria to lysosomes.^43^ In our study, the number of mito‐lysosome puncta appeared to be increased in the shOGG1 + HR24h group compared to other groups, which indicated that OGG1 could suppress the degree of mitophagy and regulate the transfer of PINK1 into mitochondria in IRI.

The PINK1–PRKN/PARK2‐mediated mitophagy pathway may be the main mitophagy pathway in renal IRI.^44^ Our results also demonstrated the repressed activation of autophagic lysosomes after PINK1‐knockdown (Figure 7G,H). Bioinformatics analysis suggested an important correlation between PINK1 and OGG1. However, due to insufficient datasets of OGG1 KO mouse kidneys, we chose the OGG1 KO dataset (GSE35497), which contains gene expression in normal chow or HFD‐fed OGG1^−/−^ mouse livers, which may interfere with the results. To explore the association between OGG1 and PINK1 in ischemic renal injury, we isolated the mitochondria and cytoplasm to examine the protein levels of PINK1. We found that PINK1 expression increased in the mitochondria after HR24h, and this increase was further enhanced by OGG1 knockdown, suggesting that OGG1 may negatively regulate PINK1. This is in agreement with our results showing increased levels of the PINK1 mitophagy pathway proteins PINK1, Parkin, and LC3 in response to HR in the BUMPTs model and a further increase when OGG1 function was inhibited or OGG1 was knocked down (Figures 8C–G and 9A–F). Consistently, fluorescence colocalization results showed that OGG1 and PINK1 were partially colocalized in the control group (yellow signal), whereas there were fewer colocalization sites in the HR24h group (yellow signal) (Figure 6G). In addition, the co‐IP experiment verified that OGG1 interacted with PINK1; however, interaction was reduced upon HR (Figure 6H). The above findings indicate that OGG1 could regulate the mitophagy pathway by interacting with PINK1 and inhibiting PINK1 translocation to mitochondria.

In summary, this study provides substantial evidence indicating OGG1 activation in ischemic renal injury and alleviation of renal IRI by OGG1 inhibition or gene KO. Furthermore, mitophagy was induced in renal tubular cells during ischemic AKI and was regulated mainly by the PINK1/Parkin pathway. In addition, OGG1 can inhibit mitophagy by binding to PINK1 and regulating the transfer of PINK1 into mitochondria in IRI. The useful improvement effect of renal IRI of the OGG1 specific inhibitor Th5487 has been verified. Therefore, OGG1 appears to be a novel clinical target with therapeutic potential.

## AUTHOR CONTRIBUTIONS

Fan Zhao, Jiefu Zhu, and Xiongfei Wu contributed to the conception of the study. Lang Shi, Jiefu Zhu, Shengyu Dong, Yuzhen Li, and Jing Huang performed the experiments. Xiongfei Wu and Jiefu Zhu contributed significantly to the analysis and manuscript preparation. Fan Zhao and Jiefu Zhu performed the data analyses and wrote the manuscript. Fan Zhao, Yanwen Luo, Juan Wang, Halinuer Shadekejiang, Mingjiao Zhang, and Shengyu Dong helped perform the analysis with constructive discussions. Jiefu Zhu and Xiongfei Wu have reviewed the manuscript.

## CONFLICT OF INTEREST STATEMENT

The authors declare no conflict of interest.

## Supporting information


**DATA S1.** Supporting Information

## Data Availability

Data available on request from the authors.
